# Subarachnoid hemorrhages and aneurysms during the SARS-CoV2-pandemia at a tertiary medical center – Analysis of incidence and outcome

**DOI:** 10.1016/j.bas.2023.101757

**Published:** 2023-05-11

**Authors:** Konstantinos Lintas, Stefan Rohde, Gisa Ellrichmann, Boris El-Hamalawi, Robert Sarge, Oliver Müller

**Affiliations:** a Neurosurgical Department, Dortmund Hospital, Germany; b Radiological and Neuroradiological Department, Dortmund Hospital, Germany; c Neurological Department, Dortmund Hospital, Germany

**Keywords:** Symptomatic aneurysms, Subarachnoid hemorrhage, N.I.N.E., Corona pandemic

## Abstract

**Introduction:**

During the COVID-19-pandemic a significant decrease of up to 13% of all kinds of medical emergencies was reported. Similar trends were expected for aneurysmal subarachnoid hemorrhages (aSAH) and/or symptomatic aneurysms.

**Research question:**

To analyze a correlation of the SARS-CoV2-infection and the incidence of aSAH, and to assess the impact of the pandemic-lockdown on the incidence, the outcome and the course of patients suffering from aSAH and/or aneurysms.

**Material and methods:**

From March 16th, 2020 (first lockdown in Germany) to January 31st, 2021, all patients admitted to our hospital were screened by polymerase-chain-reaction (PCR) test for genetic material of SARS-CoV2. During this period, aSAH and symptomatic cerebral aneurysms were assessed and retrospectively compared to a historic longitudinal case-cohort.

**Results:**

Of 109.927 PCR-tests, 7.856 (7.15%) revealed a SARS-CoV2-infection. None of the patients mentioned above were tested positively. The number of aSAH and symptomatic aneurysms rose by 20.5% (39 vs. 47 cases) (p ​= ​0.93). Poor grade aSAH, as well as extensive bleeding-patterns were more often observed (p ​= ​0.63 and p ​= ​0.40, respectively), with more symptomatic vasospasms diagnosed (5 vs. 9 patients). Mortality rate increased by 8,4%.

**Discussion and conclusion:**

A correlation between SARS-CoV2-infection and the incidence of aSAH could not be established. Still, the overall number and the number of poor-grade aSAHs increased as well as symptomatic aneurysms during the pandemic. Therefore, we might conclude that dedicated neurovascular competence should be retained in designated centers to care for these patients even or especially in special situations affecting the global healthcare system.

## Highlights

• First long-term analysis of subarachnoid hemorrhages and aneurysms in the pandemic.
• Increase of patients with poor SAH (H&H 5, Fisher-scale 4).
• Symptomatic vasospasm occurred 1.6 times more frequently.
• Importance to maintain a neurovascular and neurointensive competence.

## Non-standard abbreviations and acronyms:

COVID-19: coronavirus disease 2019
SARS-CoV-2: severe acute respiratory syndrome coronavirus type 2
DSG: German Stroke Society
TIAs: transient ischemic attacks
PCI: percutaneous coronary interventions
aSAH: subarachnoid hemorrhages
H&H: Hunt and Hess
EVD: external ventricular drain
mRS: modified Ranking Scale
ICU: intensive care unit
N.I.N.E: non-ischemic neurovascular emergencies
GSNR: German Society of Neuroradiology
GSN: German Society of Neurosurgery
DSG: German Stroke Society

## 1 Introduction

A COVID-19-infection might affect the central nervous system as well as its vessels with an inflammatory response (RKI Bulletin, 2021). Neurological symptoms with a COVID19-infection were reported in about 51% (Soltani et al., 2021). Associated ischemic and/or hemorrhagic strokes have been described (Leven and Bösel, 2021). Therefore, the possible direct influence of a COVID19-infection as well as a potential indirect impact of the lockdown - due to the pandemic - on the incidence of aneurysmal subarachnoid hemorrhage (aSAH) and symptomatic aneurysms treated is of special interest to treating physicians.

However, little information is found on either topic. While the pandemic and the lockdown affected the clinical practice in neurovascular centers in Germany as well as worldwide, a specific impact was sparsely reported on the decline of the incidence of aneurysmal subarachnoid hemorrhages treated (SVIN COVID-19 Global SAH Registry, 2022), (Anna et al., 2020). (Luostarinen et al., 2020), (Miękisiak et al., 2022). Non-aneurysmal subarachnoid hemorrhage accompanying a COVID19-infection is described only occasionally (Ersegun Batcik et al., 2021).

Publications of the German Stroke Society (DSG), as well as the German Cardiac Society, revealed that a profound decrease in heart attacks and strokes was noted during the first lockdown, that lasted from March 16^th^ 2020, until approximately January 31st^,^ 2021.

While a remarkable decline in catheter activity of 35% was noted, at the same time an increase of cardiac or cardiovascular mortality of 8%, and 12%, respectively, was found. (NEF Clin Res Card, 2021) This observation was confirmed by the results of the large multicenter ISACS-STEMI COVID-19 registry showing that significantly fewer percutaneous coronary interventions (PCI) were carried out in European centers during the first corona wave compared to the previous year (18.9%, p ​< ​0.0001), (Pessoa-Amorim et al., 2020).

The same applied for acute ischemic strokes, where the DSG reported a decline in hospitalization by 17.4% during the first couple of months of lockdown. Both, patients suffering from mild symptoms (transient ischemic attack (TIA)), as well as those with severe strokes were less likely to be treated at hospitals with numbers falling to almost 23%, and nearly 17%, respectively. These data were collected from 1463 hospitals in Germany (Richter et al., 2021).

However, a longitudinal case-cohort study, performed at our tertiary medical referral center, showed an increase of 29% in number and severity of non-ischemic neurovascular emergencies (NINE), as well as on the incidence of neurosurgical traumata during the pandemic (Lintas et al., 2022), (Lintas et al., 2022).

It is the aim of the study to investigate on the one hand a possible coincidence of a COVID19-infection and the occurrence of aneurysmal subarachnoid hemorrhages, and on the other hand the impact of pandemic and the lockdown on the incidence of aneurysmal subarachnoid hemorrhages and patients with symptomatic aneurysms treated at a third-level medical referral center and maximum care provider.

## 2 Methods

### Ethical approval

The study protocol was approved by the ethic committee of the medical association of Westfalen-Lippe (2021-687-f-S). The study was carried out under the guidelines of Good Clinical Practice according to the WMA principles of the declaration of Helsinki.

### 2.1 Patients’ selection

The present study analyzes of the medical records of all patients who were treated for an aneurysmal subarachnoid hemorrhage and/or a symptomatic aneurysm during the pandemic period in a defined time frame from March 16th, 2020 to January 31st, 2021. A longitudinal control cohort was defined from the same time period 2019–2020. Patients suffering from non-aneurysmal subarachnoid hemorrhage were excluded from the study. Asymptomatic aneurysm treatments were excluded from analysis, as well.

### 2.2 Detecting SARS-CoV-2 infection (COVID19)

All patients admitted to the hospital were screened for SARS-CoV2-infection by polymerase chain reaction (PCR) test.

### 2.3 Definition of ‘symptomatic aneurysm’

Aneurysms, that either had bled, caused (progressive) neurological deficits or had grown substantially (>1 ​mm in any direction on follow-up regardless of the underlying time frame) were defined symptomatic.

### 2.4 Scores and grading scales

At the admission of the patient, the Hunt & Hess (H&H) scale was used as a grading system to classify the severity of a subarachnoid hemorrhage based on the patient's clinical condition and the bleeding patterns in the radiological diagnostic were classified according to the Fisher scale. The outcome at discharge of the patients were scored using the modified Rankin Scale (mRS).

### 2.5 Definition of symptomatic vasospasm/delayed cerebral ischemia

Symptomatic vasospasm is defined as clinical deterioration attributable to vasospasm after other possible causes (eg, hydrocephalus, cerebral edema, seizures, electrolytes abnormality, infection, etc) had been excluded (Frontera et al., 2009), (Suwatcharangkoon et al., 2022).

A symptomatic vasospasm was diagnosed by neurologic examination and/or daily Doppler examination with an accelerated blood vessel flow that either transcended >190 ​cm/s, or increased by at least 50% of the velocity recorded prior. In cases of uncertainty a CT-perfusion scan was complemented. After the verification, a digital subtraction angiography with subsequent intra-arterial spasmolysis was performed.

### 2.6 Data analysis

This is a retrospective, non-randomized, non-blinded and non-placebo-controlled data analysis at a tertiary medical center in Germany. The hospital is situated in an area inhabited by approximately seven million citizens (DESTATIS, 2021). Six tertiary referral centers with neurosurgical facilities serve this region. It is a dedicated specialized clinic for neurovascular diseases, board certified by DSG, DGNR and DGNC.

Statistical analysis was performed employing Excel 16.50 (Microsoft® Office, Excel®, 2021), using descriptive analysis and Chi-square-test for standard normal distribution. Statistical differences in categorical variables between patients were calculated using χ2 test and for continuous variables using *t*-test. An α-mistake of p ​< ​0.05 was defined as statistical significance.

All data of the present study are available upon request from the corresponding author upon reasonable intention.

## 3 Results

### 3.1 Study cohorts

A total of 1669 admissions were screened for study purposes. Between March 16, 2019 and January 31, 2020 (pre-defined period 1), 764 neurosurgical emergencies were admitted. Of these, 39 were diagnosed with aSAH and/or symptomatic aneurysms according to the criteria given above. During the period of the lockdown (pre-defined second period), from March 16th, 2020 to January 31st, 2021, a total of 905 neurosurgical patients were referred to the neurosurgical department. Among them, 47 patients were diagnosed with aSAH and/or symptomatic aneurysms requiring urgent professional attendance (Table 1 and Table 2).

**Table 1:** Patients with SAH und symptomatisch Aneuryms

	period 1 (cohort-control group, March 16th, 2019–January 31st, 2020)	period 2 (Corona pandemic, March 16th, 2020–January 31st, 2021)
female patients	20	34
male patients	19	13
mean age (years)	54,1	59,4
duration total hospital stay (days)	1136	1089
duration in ICU (days)	848	879
duration in peripheral wards (days)	288	210
patients primary admitted at our hospital (n)	13	12
patients that were transferred from other hospitals (n)	26	35

**Table 2:** Comorbidities.

	period 1 (cohort-control group, March 16th, 2019–January 31st, 2020)	period 2 (Corona pandemic, March 16th, 2020–January 31st, 2021)
cardiovascular diseases	3	2
arterial hypertension	7	7
combination of more than one system diseases	5	10
none	24	28

Within both periods, a total of 86 patients meeting the inclusion criteria were treated. The absolute number of cases increased by 20.5% during the lockdown. Still, the increase was statistically not significant within the standard distribution (chi2 p ​= ​0.53). The patients’ cohorts were comparable regarding gender and age, with a slight female preponderance, within both time periods.

Of note, the total number of severe emergencies others than neurovascular (defined as polytraumatized patients) referred to our tertiary medical center decreased by 10% during the pandemic (n ​= ​252 in 2019–20; n ​= ​226 in 2020–21).

### 3.2 SARS-CoV-2 infection (COVID19)

Screening all admitted patients of the hospital, a total of 109.927 PCR tests were done during the study period. Of these, 7.856 PCR tests (7.15%) detected genetic materials of SARS-CoV2. There were no SARS-CoV2 infections found among patients with aSAH and/or symptomatic aneurysms in the study cohort.

### 3.3 Scores and grading scales

**Fig. 1:**
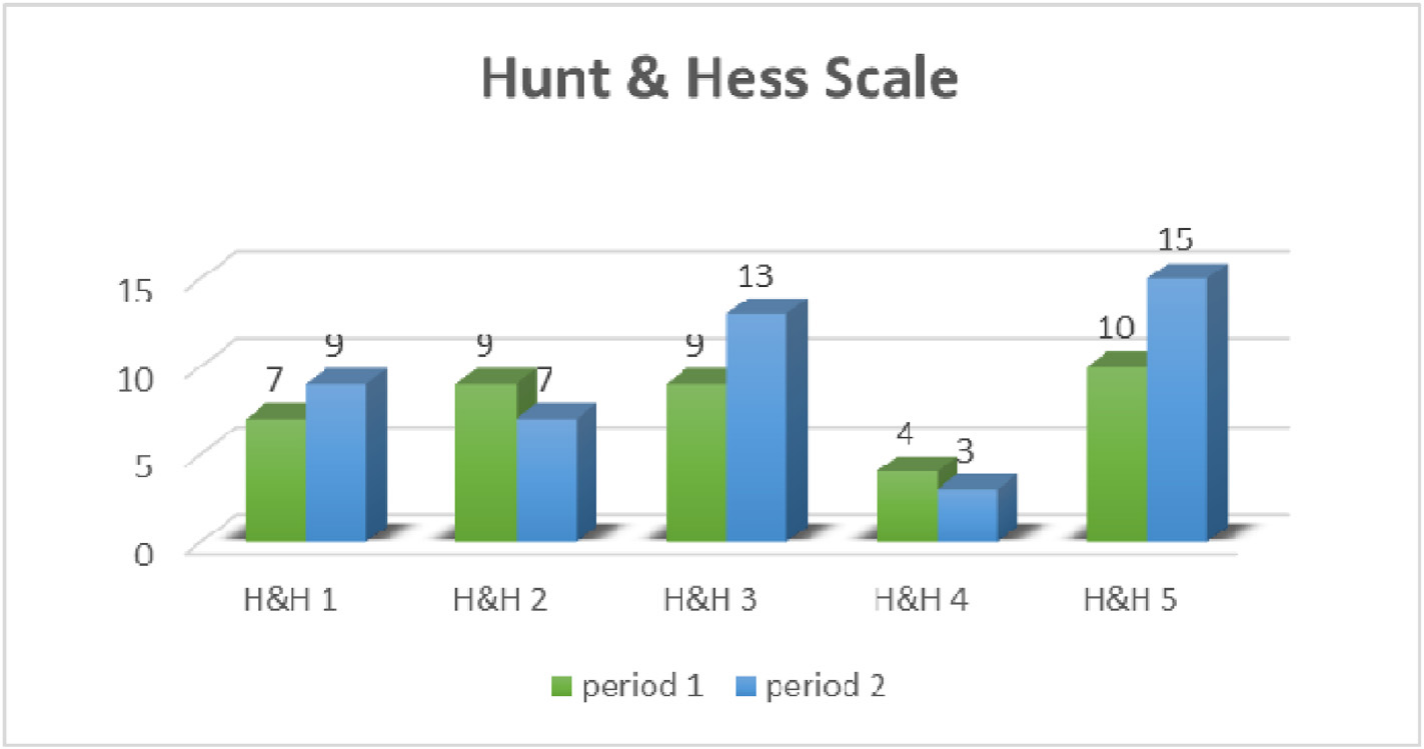
legend: Hunt & Hess Scale. Patient during March 16th, 2019–January 31st, 2020 were included for period 1 data (green column), period 2 was defined from March 16th, 2020 to January 31st, 2021 (blue column). (For interpretation of the references to colour in this figure legend, the reader is referred to the Web version of this article.)

Patients with light to moderate affection due to aSAH were equally distributed in both cohorts. There was an increase in severely (H&H3) and heavily (H&H5) affected patients by 50%, though not reaching statistical significance ((p ​= ​0.63), for details see Fig. 1).

**Fig. 2:**
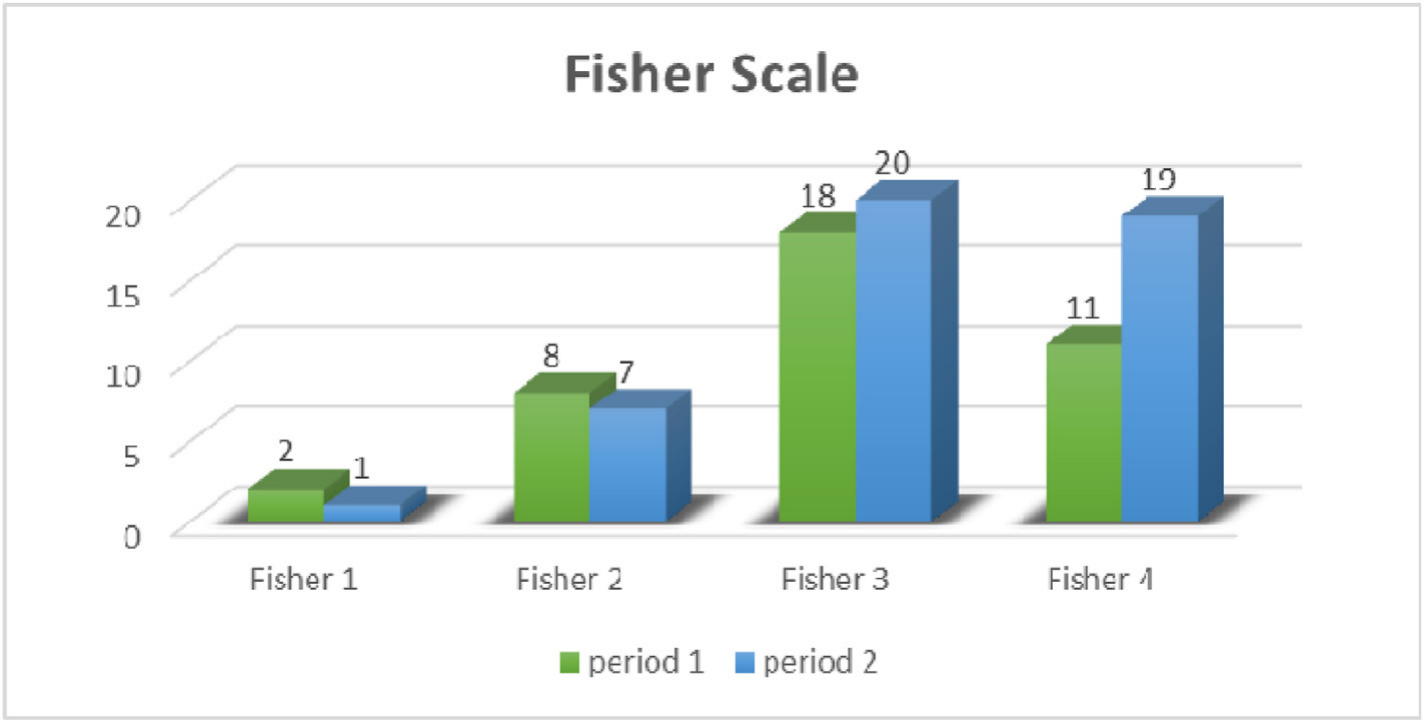
legend: Fisher Scale. Patient during March 16th, 2019–January 31st, 2020 were included for period 1 data (green column), period 2 was defined from March 16th, 2020 to January 31st, 2021 (blue column). (For interpretation of the references to colour in this figure legend, the reader is referred to the Web version of this article.)

Likewise, aSAH with bleeding patterns according to Fisher scale 4 rose substantially in number by 72.7%. Still, this increase was within the normal distribution not reaching statistical significance (p ​= ​0.40), for details Fig. 2.

**Fig. 3:**
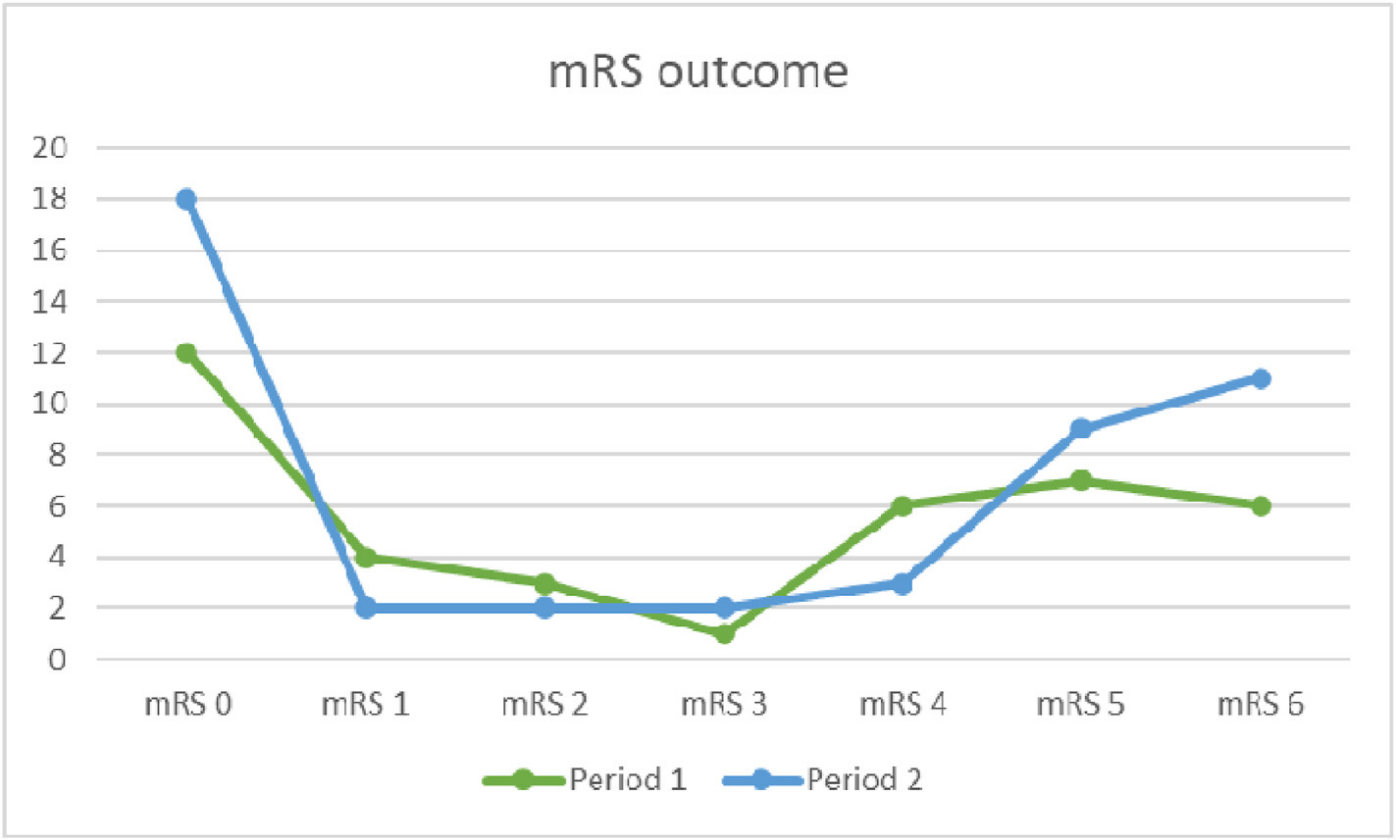
legend: Outcome at discharge of patients in both time periods using the modified Rankin Scale (mRS). All patients were scored using the modified Rankin Scale (mRS). The scale runs from 0 to 6: 0 – no symptoms, 1 – no significant disability, able to carry out all usual activities, despite some symptoms, 2 – slight disability, able to look after own affairs without assistance, but unable to carry out all previous activities, 3 – moderate disability, requires some help, but able to walk unassisted, 4 – moderately severe disability, unable to attend to own bodily need without assistance, and unable to walk unassisted, 5 – severe disability, requires constant nursing care and attention, bedridden, incontinent, 6 – dead.Patient during March 16th, 2019–January 31st, 2020 were included for period 1 data (green line), period 2 was defined from March 16th, 2020 to January 31st, 2021 (blue line).There was an increase of number of patients with fatal outcomes during the pandemic period (period 2) resulting in higher numbers of patients with mRS 5–6. (For interpretation of the references to colour in this figure legend, the reader is referred to the Web version of this article.)

During time period 2, patients had a worse outcome with respect to the modified Ranking Scale (mRS). Here, fatal outcomes increased by in numbers by 83% (6 deaths vs. 11 deaths). In accordance, the mortality rate raised during the second period (15% vs 23.4%), due to the overall limited sample size not reaching statistical significance (p ​= ​0.44) (Fig. 3).

Contrarily, we observed an increase of patients leaving the hospital in excellent condition (mRS 0) during the lockdown, too. Thus, the increase of case-load in the second cohort was not caused by a raised incidence of severely afflicted patients, solely.

### 3.4 Analysis of neurovascular pathologies

I) Symptomatic vasospasm/delayed cerebral ischemia analysis

Incidence of symptomatic vasospasms did not differ significantly between cohorts (period 1: 12% vs. period 2: 19%). There were more cases detected in period 2 (5 vs. 9 cases), yet not reaching statistical significance. Vasopasms were confirmed by Doppler examination and DSA in all events. All patients were treated with intraarterial spasmolysis.

II) Aneurysms’ localisation

**Fig. 4:**
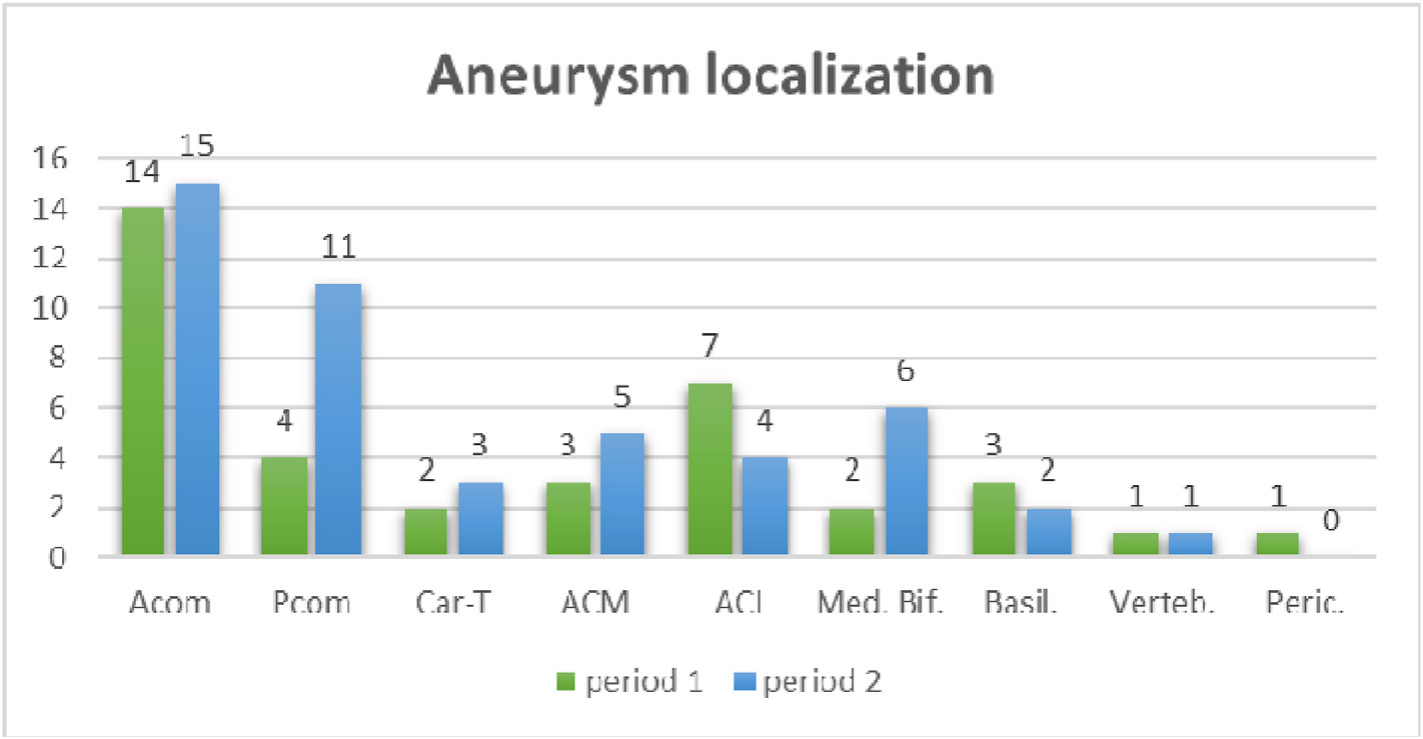
legend: Aneurysm localisation. Patient during March 16th, 2019–January 31st, 2020 were included for period 1 data (green column), period 2 was defined from March 16th, 2020 to January 31st, 2021 (blue column). (For interpretation of the references to colour in this figure legend, the reader is referred to the Web version of this article.)

Aneurysms of the circle of Willis were equally distributed in both cohorts with respect to their localisation. Affected vessels carrying symptomatic aneurysms are displayed in Fig. 4 for both cohorts.

III) Associated hydrocephalus

The number of patients suffering from post-hemorrhagic hydrocephalus in need of cerebro-spinal fluid (CSF) diversion (exclusively external ventricular drainage, EVD) grew in number during the second period (period 1: n ​= ​26 vs period 2: n ​= ​34), too, without reaching statistical significance (p ​= ​0.80). Furthermore, we observed an increase of patients in need of a permant CSF-diversion by 50% during the pandemic. Still, this increase, too, was within the normal distribution not reaching statistical significance (p ​= ​0.635).

IV) Treatment modality

**Fig. 5:**
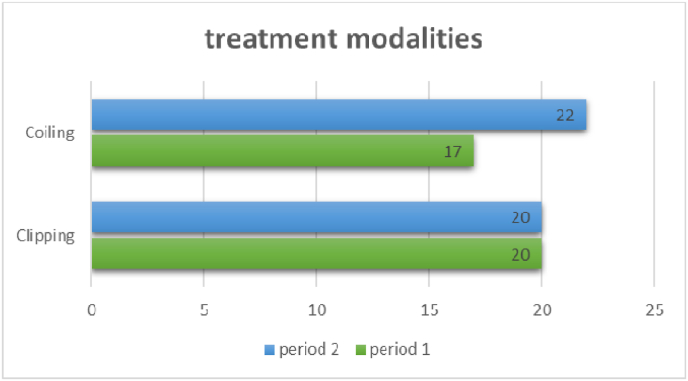
legend: Treatment modalities (clipping and coiling). Patient during March 16th, 2019–January 31st, 2020 were included for period 1 data (green column), period 2 was defined from March 16th, 2020 to January 31st, 2021 (blue column). (For interpretation of the references to colour in this figure legend, the reader is referred to the Web version of this article.)

With respect to treatment modalities, a slight increase of cases treated endovascularly by 3.8% (43% vs 46.8%) was noted during the second time period (Fig. 5).

The individual duration of total hospital stay decreased by 6 days during the second period (29.1 days/patient, vs. 23.1 days/patient). Neither the total days of hospitalization, nor the length of stay on the intensive care unit (848d vs. 879d) differed significantly.

## 4 Discussion

The SARS-CoV2 infection did not have an impact on the incidence neither of aSAH nor symptomatic aneurysms, according to the present data. Even though, one could have assumed that an involvement of the CNS, especially of its vessels, in COVID 19 diseases could have led to a raise in aSAH, this hypothesis is dismissed by the findings of this study.

This is the first detailed analysis of the impact of the pandemic and the following lockdown on incidence, severity and short-term outcome of patients suffering from aSAH and symptomatic aneurysms.

From what is reported in the literature so far, it was expected that number of patients would decrease in a similar fashion to non-hemorrhagic stroke or heart attacks. This assumption was supported by a recent publication of Nguyen et al., who reported on a decrease of aSAH in a global cohort over a three-months-period within the same time frame (Nguyen et al., 2021). Furthermore, a decrease in hospitalization of patients with aSAH could be noticed on a global scale during the pandemic (Nguyen et al. , 2021). Numerous publications have consistently reported that the total number of hospital admissions across all disciplines have markedly declined during the pandemic (Birkmeyer et al, 2020), (Kapsner et al., 2020), (Nourazari et al., 2021). This finding is supported by this study as a marked decrease of emergencies others than cerebrovascular events was recorded (10%)

Contrarily, we observed an increase in the admittance of patients suffering from aSAH and symptomatic aneurysms in the study cohort.

This finding might be explained by several circumstances. First, the social distancing presumably led to reduced interaction of patients with family and friends, with early and mild symptoms of any disease not being recognized. Interaction with relatives, mainly non-physicians, has been identified as one keystone in the decision-making process to seek professional medical help, even though it may even delay treatment (Mahmud et al., 2020). Therefore, it is possible that early warning signals might have been missed, leading to urgent admission with progressive symptoms.

Second, patients avoided consulting their family physician, because of they were afraid of getting infected with SARS-CoV2 when getting in close contact with other patients. Concurrently, many physicians reduced direct contact to patients, rather preferring consultation by phone. Thus, emerging warning signs - red-flags - indicating an uprising severe condition were most likely to be overlooked. It has been reported that patients generally tend to see their practitioner before going to an emergency or outpatient department at a hospital (Gaertner et al., 2008).

Last but not least, smaller hospitals shut down their emergency departments. It can only be speculated that this led to a partial regional reorganisation of transportation pathways for emergencies seeking neurosurgical attendance. Whether this led to a delayed presentation of distinct cases to the neurosurgical department leading to the observed increase of fatal outcomes is beyond the scope of this study.

There was an unambiguous decrease of the individual duration of total hospital stay during the second period. Personal communication with the rehabilitation facilities endorsed, that most of them were unable to take over patients in compromised conditions due to a lack of vacancies and nursing staff during the lockdown. Therefore, patients were deprived of rehabilitation and rather discharged home, whenever possible.

Our study has several limitations. A major limitation of this study is its monocentric retrospective nature. Though not obvious, regional factors could have influenced the admission rates and/or transfer of patients with aneurysms to our neurovascular center. Furthermore, the present study observes a distinct period of time during the COVID pandemic. It remains uncertain whether the vaccination status of the general population or the relaxation of the lockdown restrictions would have an impact on further study results.

## 5 Conclusion

A SARS-CoV2-infection is not associated with an increased risk of aSAH. The management and the outcome of patients suffering from aSAH during the lockdown is equal to comparable periods prior to the pandemic. Probably, the outcome might be slightly diminished, due to more severely affected patients in this series.

From our data we may conclude that it seems of utmost importance for the care of patients harboring neurovascular disease to retain distinct neurosurgical capabilities, and further applies to retain neurointensive care capacities.

In addition, our data strengthens the need to maintain a dedicated neurovascular competence in designated centers to care for these non-ischemic neurovascular emergencies (N.I.N.E.) even in special times as the COVID pandemic.

## Sources of funding

None.

## Disclosures

None.

## Declaration of competing interest

The authors declare that they have no known competing financial interests or personal relationships that could have appeared to influence the work reported in this papesr.
